# Clinical utility of targeted next‐generation sequencing for the diagnosis of myeloid neoplasms with germline predisposition

**DOI:** 10.1002/1878-0261.12921

**Published:** 2021-07-16

**Authors:** Cristina Andrés‐Zayas, Julia Suárez‐González, Gabriela Rodríguez‐Macías, Nieves Dorado, Santiago Osorio, Patricia Font, Diego Carbonell, María Chicano, Paula Muñiz, Mariana Bastos, Mi Kwon, José Luis Díez‐Martín, Ismael Buño, Carolina Martínez‐Laperche

**Affiliations:** ^1^ Genomics Unit Gregorio Marañón General University Hospital Gregorio Marañón Health Research Institute (IiSGM) Madrid Spain; ^2^ Gregorio Marañón Health Research Institute (IiSGM) Madrid Spain; ^3^ Department of Hematology Gregorio Marañón General University Hospital Madrid Spain; ^4^ Department of Medicine School of Medicine Complutense University of Madrid Spain; ^5^ Department of Cell Biology School of Medicine Complutense University of Madrid Spain

**Keywords:** family history, genetic counseling, hereditary cancer, myeloid neoplasms with germline predisposition, next‐generation sequencing

## Abstract

Myeloid neoplasms (MN) with germline predisposition (MNGP) are likely to be more common than currently appreciated. Many of the genes involved in MNGP are also recurrently mutated in sporadic MN. Therefore, routine analysis of gene panels by next‐generation sequencing provides an effective approach to detect germline variants with clinical significance in patients with hematological malignancies. Gene panel sequencing was performed in 88 consecutive and five nonconsecutive patients with MN diagnosis. Disease‐causing germline mutations in *CEBPα, ASXL1, TP53, MPL, GATA2, DDX41*, and *ETV6* genes were identified in nine patients. Six out of the nine patients with germline variants had a strong family history. These patients presented great heterogeneity in the age of diagnosis and phenotypic characteristics. In our study, there were families in which all the affected members presented the same subtype of disease, whereas members of other families presented various disease phenotypes. This intrafamiliar heterogeneity suggests that the acquisition of particular somatic variants may drive the evolution of the disease. This approach enabled high‐throughput detection of MNGP in patients with MN diagnosis, which is of great relevance for both the patients themselves and the asymptomatic mutation carriers within the family. It is crucial to make a proper diagnosis of these patients to provide them with the most suitable treatment, follow‐up, and genetic counseling.

## Graphical abstract

Germline predisposition to hematologic malignancies was historically thought to be rare; however, a widespread use of novel technologies has led to an enlarged description of familial cases and the number of mutations involved. This review discusses how the identification of an inherited predisposition syndrome allows to optimize the management of the patient and the opportunities for identification other family members at risk.

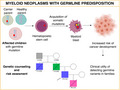

**Abbreviations**
Allo‐HSCT: allogeneic hematopoietic stem cell transplantation
AML: acute myeloid leukemia
BM: bone marrow
COSMIC: Catalogue of Somatic Mutations in Cancer
ET: essential thrombocythemia
MDS: myelodysplastic syndrome
MN: myeloid neoplasms
MNGP: myeloid neoplasms with germline predisposition
NGS: next‐generation sequencing
PB: peripheral blood
PMF: primary myelofibrosis
PV: polycythemia vera
VAF: variant allele frequency
VUS: variants of uncertain significance
WES: whole‐exome sequencing
WGS: whole‐genome sequencing
WHO: World Health Organization

## 1 Introduction

Chronic myeloproliferative neoplasms, myelodysplastic syndromes (MDS), and acute myeloid leukemia (AML) are genetically heterogeneous groups of clonal hematopoietic disorders characterized by morphological changes and ineffective hematopoiesis [1, 2] together referred to as myeloid neoplasms (MN). While MN are mainly sporadic and primarily diseases of the elderly, germline mutations contributing to MN are not well defined. However, in recent years the increasing application of next‐generation sequencing (NGS) has resulted in the recognition of multiple *loci* (*GATA2, RUNX1, CEBPα, DDX41, ETV6, ANKRD26, SRP72,* or *SAMD9*) [3, 4, 5, 6, 7] that the updated 2016 World Health Organization classification of hematopoietic tumors included as a new category named MN with germline predisposition (MNGP) [8]. It seems that they usually appear in 1–2% of elderly patients, and around 4–13% in children, young, and middle‐age adults [9]. Furthermore, the growing interest of the scientific community regarding genetic predisposition of cancer will probably result in the recognition of a higher incidence in the future.

The detection of individuals with these inherited disorders allows several opportunities for improvement in clinical care. Thus, it is important for clinicians to be familiar with the diagnosis, evaluation, and management of affected patients. A detailed family medical history, collected in all suspicious cases through the realization of a complete pedigree, should be mandatory. Once a germline mutation is confirmed in nonhematopoietic tissue, family members should be referred to genetic counseling.

Moreover, the recognition of such patients is crucial for the identification of asymptomatic carrier family members [10, 11, 12] in the donor selection process for allogeneic hematopoietic stem cell transplantation (allo‐HSCT).

For these reasons, the inclusion of new genes involved in MNGP in NGS myeloid gene panels would improve the diagnosis and identification of these disorders [2, 13]. In addition, many of these genes are also recurrently mutated in sporadic MDS/AML.

Thus, our main objective was to analyze whether employing an NGS gene panel‐based approach in MN diagnosed patients enables the identification of clinically relevant germline variants. In addition, for those patients in whom germline mutations were identified, a careful revision of the oncohematological family history was performed to characterize the disease and the underlying molecular events.

## 2 Patients and methods

### 2.1 Patient selection

**Fig. 1:**
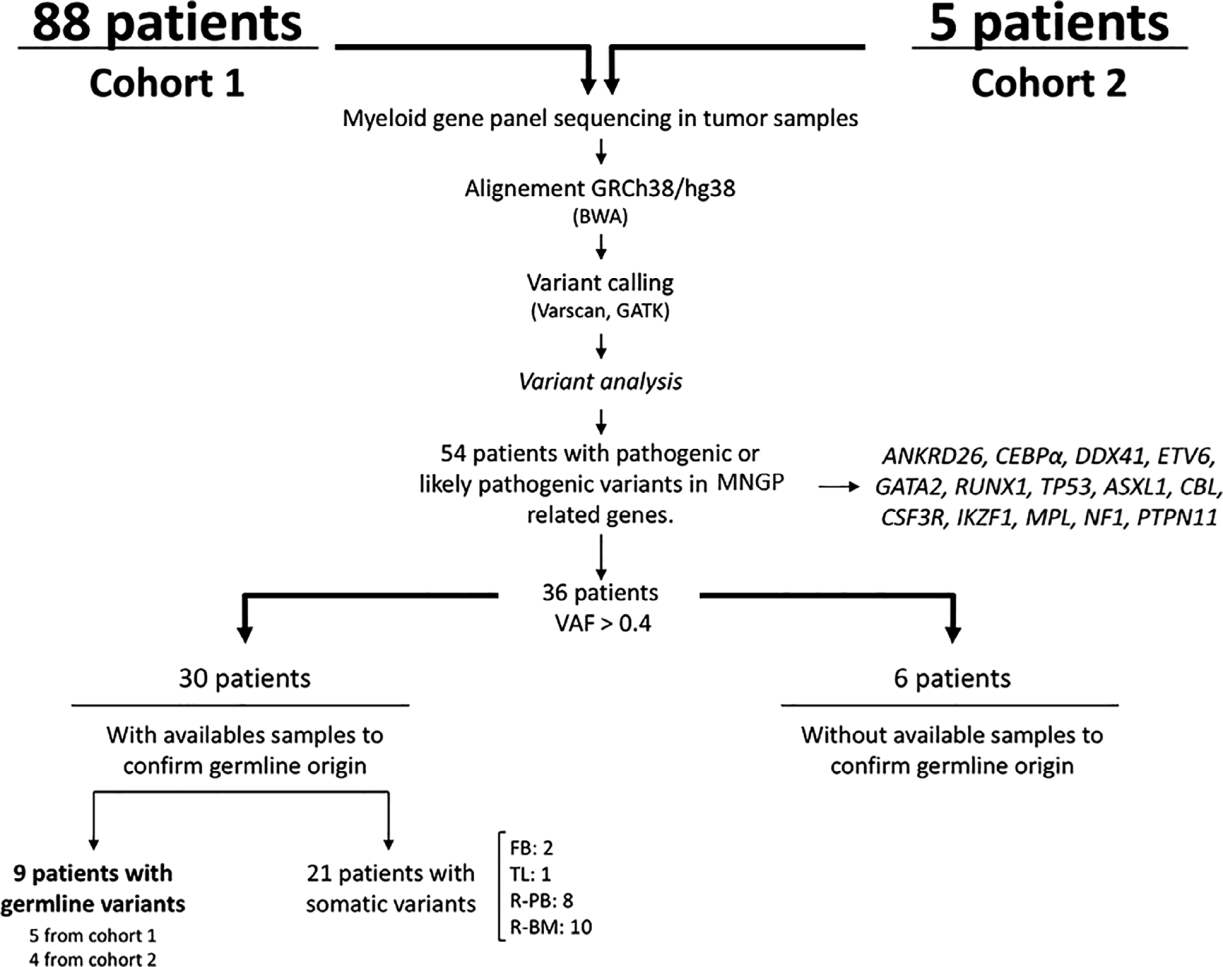
Workflow for the analysis of variants in the 93 patients studied (88 with MN diagnosis and five with suspected MNGP). BWA, Burrows‐Wheeler Aligner; FB, fibroblasts; R‐PB, remission PB; R‐BM, remission bone marrow.

A myeloid gene panel was analyzed by NGS in 88 adults with a diagnosis of MN in the hematology department of Hospital General Universitario Gregorio Marañón from May 2017 to February 2019 (Cohort 1; Fig. 1 and Table 1. Patients diagnosed with AML and primary myelofibrosis (PMF) were all included. MDS, polycythemia vera (PV), essential thrombocythemia (ET), and MDS/myeloproliferative neoplasms (MPN) patients were only included if they were candidates to receive standard therapy or eligible to participate in a clinical trial. Patients diagnosed with bone marrow (BM) failure syndromes have not been included in the present study.

**Table 1:** Clinical characteristics of the 93 patients (88 in cohort 1 and five in cohort 2) included in the study (Hb, hemoglobin).

	Cohort 1 (*n* = 88)	Cohort 2 (*n *= 5)
Sex, *n* (%)		
Female	28 (32)	3 (60)
Male	60 (68)	2 (40)
Age (years), median [range]	61 [14–82]	32 [15–45]
MN subtypes, *n* (%)		
AML	30 (34)	2 (40)
MDS	32 (36)	2 (40)
MPN	22 (25)	1 (20)
MDS/MPN	4 (5)	0 (0)
Laboratory, median [range]		
Leukocytes (× 10^9^/L)	5.6 [0.9–326.9]	3.3 [0.9–5.6]
Neutrophils (× 10^9^/L)	2.8 [0.1–109.3]	1.1 [0.2–3.1]
Platelets (× 10^9^/L)	112 [11–1049]	117 [11–200]
Hb (g·dL^−1^)	10.5 [5.9–17.6]	9 [7.2–11.4]
BM blasts (%)	10.5 [0–96]	0 [0–53]
PB blasts (%)	1 [0–98]	10 [0–36]
LDH (U·L^−1^)	181 [74–428]	201.5 [123–280]
Karyotype, *n* (%)		
Normal	34 (39)	0 (0)
Altered	25 (28)	1 (20)
Complex	9 (10)	1 (20)
No karyotype	20 (23)	3 (60)
Molecular alterations, patients (%)		
*DNMT3A*	10 (11)	0 (0)
*RUNX1*	9 (10)	1 (20)
*NPM1*	8 (9)	0 (0)
*FLT3*	5 (6)	0 (0)
*IDH1*	2 (2.3)	0 (0)
*IDH2*	2 (2.3)	0 (0)

Furthermore, five patients with a high suspicion of having MNGP diagnosed from 2010 to 2016 were also included (Cohort 2; Fig. 1 and Table 1. Patients were considered highly suspicious when they had (a) more than one first‐degree relative affected from hematological malignancies or other solid tumors, (b) organ‐system manifestations fitting known MNGP, and/or (c) personal history of multiple oncohematological tumors.

The study was conducted in accordance with the Declaration of Helsinki, and ethical approval was obtained by the Ethical Committee of University Hospital Gregorio Marañón. Personal and family histories, demographics, and patient characteristics were obtained from the electronic health records.

### 2.2 Analysis of molecular alterations through NGS

Genomic DNA was purified from BM aspirates or peripheral blood (PB) samples at diagnosis following the manufacturer's instructions (Maxwell® 16 Blood DNA Purification Kit; Promega, Madison, WI, USA).

NGS‐based targeted gene capture panel (*MyeloidNeoplasm‐GeneSGKit*; Sistemas Genómicos, Valencia, Spain. Table S1) is routinely performed in our hospital to characterize MN patients [14]. Consistent with the current knowledge, the panel included 15 genes known to be involved in MNGP (*ANKRD26, ASXL1, CBL, CEBPα, CSF3R, DDX41, ETV6, GATA2, IKZF1, JAK2, MPL, NF1, PTPN11, RUNX1, and TP53*) [8, 15, 16, 17, 18, 19, 20]. Target enrichment experiments were performed according to standard manufacturer's protocol for library preparation. Probes were designed to detect point mutations, *indels* < 30 base pairs, copy number variations, chromosomal rearrangements, and numerical alterations. Libraries were denatured and sequenced on an Illumina MiSeq platform with reagents v2 for paired‐end sequencing (2*101 bp).

FASTQ files were aligned to the human genome reference sequence (GRCh38/hg38) using Burrows‐Wheeler Aligner [21] and ‘in‐house’ scripts. Variant calling was conducted using a combination of two different algorithms: VarScan [22] and GATK [23]. Identified variants were annotated using Ensembl database, population databases (the Exome Aggregation Consortium and 1000 Genomes), and specific variant databases (ClinVar, Catalogue of Somatic Mutations in Cancer (COSMIC), Online Mendelian Inheritance in Man, and Human Gene Mutation Database). Variants were evaluated with Polyphen 2.0, SIFT, and Mutation Taster softwares to predict their functional effects.

### 2.3 Variant analysis

After annotation, all frameshift and nonsynonymous variants located in coding or splicing regions of canonical isoforms, affecting all genes included in the panel and with minor allele frequency < 1%, were considered in subsequent analyses. Additionally, *GATA2* synonymous variants were carefully analyzed, as recently reported [24, 25]. The pathogenicity of variants was designated according to the recommendation of the American College of Medical Genetics and Genomics and the Association for Molecular Pathology Fig. S1 [26].

Pathogenic or likely pathogenic variants were suspected to be of germline origin if they showed an allelic frequency [variant allele frequency (VAF)] > 0.4 for single nucleotide variations and > 0.3 for small insertions or deletions (indels). In such cases, variants may be either of heterozygous germline or of acquired origin. As the panel employed is capable to detect copy number alterations reliably, all cases were also carefully analyzed in order not to miss any potential germline variant with lower VAF than expected.

### 2.4 Confirmation of germline origin

As a first approach, PB/BM samples at complete remission (CR) or CD3^+^ T cells were employed to evaluate the origin of the mutation. Immunomagnetic separation method was carried out for the purification of T lymphocytes (TL) using anti‐CD3 MicroBeads (Miltenyi Biotec 130‐050‐101, Bergisch Gladbach, Germany). Cell selection was performed on the auto‐MACS PRO Separator (Miltenyi Biotech) using two paramagnetic columns, and positive selection was then retained.

With the purpose of definitely confirming germline origin, a skin biopsy sample was requested to carry out skin fibroblast culture if possible. Skin fibroblast culture was conducted at 37 °C, in humidity conditions, 5% CO_2_, and in Roswell Park Memorial Institute (RPMI) cell culture media (Gibco, Thermo Fisher Scientific, Waltham, MA, USA) supplemented with glutamine, FBS, and 1% of penicillin and streptomycin during 4–6 weeks. After this time, cells were trypsinized and DNA was extracted using QiAmp DNA Mini Kit (Qiagen, Hilden, Germany) according to manufacturer's instructions.

The confirmation was performed by optimizing specific PCR assays for each variant followed by Sanger sequencing with Big Dye Terminator v3.1 Chemistry (Applied Biosystems, Thermo Fisher Scientific, Waltham, MA, USA) on ABI 3130xl or ABI 3730xl Genetic Analyzer (Applied Biosystems). Visualization and localization of variants were assessed by using chromas Software (Technelysium, South Brisbane, Australia) and the Basic Local Alignment Search Tool (BLAST) (Bethesda, MD, USA).

Once a germline variant was confirmed in the patient, a segregation study was offered to his relatives. In cases in which a rapid answer was needed from the laboratory (i.e., patient candidate to HSCT), the variant was analyzed in a nontumoral sample at the same time as potential family donors were being evaluated. In the rest of cases, we first screened the mutation in remission samples or TL and, if positive, a skin biopsy was requested when it was feasible.

## 3 Results

### 3.1 Variant analysis

Regarding cohort 1, a total of 329 variants were detected, of which 62% (205/329) were classified as pathogenic or likely pathogenic (P/LP), 33% (108/329) were classified as variants of uncertain significance (VUS), and 5% (16/329) were classified as benign or likely benign (B/LB). Taking into account only the 15 genes of potential germline relevance, 133 variants were detected. Among them, 45% (60/133) were classified as P/LP, 46% (61/133) were classified as VUS, and 9% (12/133) were classified as B/LB. Thus, 32 patients carried pathogenic or likely pathogenic variants with a VAF > 0.4 in these 15 genes. We could study the possible germline origin of 27 variants in 26 patients Table S2, and the remaining six patients could not be analyzed because nontumoral sample was not available. After confirmatory tests, five patients were confirmed of harboring germline variants.

Thus, in cohort 1 a total 5.7% (5/88) of patients presented MNGP. The affected genes were *MPL* (p.Phe105Leu), *GATA2* (p.Arg396Gln), *DDX41* (p.Asp30_Asp32del; p.Ile592Phe), and *ETV6* (p.Arg49Cys) Tables 2 and 3. Interestingly, in all cases suspected with germline variants in *MPL, DDX41,* and *ETV6* genes, these were finally confirmed as of germline origin Tables 2 and 3.

**Table 2:** Relevant clinical characteristics of individuals affected from MNGP in the studied families. Dash indicates not chemotherapy received. Aza, azacitidine; Ch, chemotherapy; F, female; Fam, family; Ind, individual; lena, lenalidomide; M, male; MMS, Mono‐Mac syndrome; NA, not available; NT, not treated; pred, prednisone.

Fam	Ind	Cohort	Sex	Age (years)	Gene	Diagnosis	BM blasts (%)	Karyotype	Ch	Status	HSCT	Follow‐up (months)
A	III.1	2	F	32	*CEBPα*	AML	53	47,XX,+21 [18/20]; 46,XX [2/20]	IA 3X7	CR	No	132
A	II.1		F	53	*CEBPα*	AML	NA	46,XX [10]	IA 3X7	CR	auto‐HSCT	147
B	IV.3	2	F	2	*ASXL1*	PMF	NA	46,XX [20]	Lena + pred	CR	No	220
B	III.6		M	54	*ASXL1*	PMF	NA	NA	Lena	Progression	No	88
C	III.6	1	F	53	*ETV6*	MDS	6	46,XX [20]	NT	Progression	No	100
C	II.6		F	86	*ETV6*	MDS	2	46,XX [20]	NT	*Exitus*	No	45
D	II.1	1	M	41	*TP53*	AML	89	Complex	IA 3X7	*Exitus*	No	14
E	II.2	1	M	53	*MPL*	MPN	1	46,XY [20]	NT	Alive	No	18
F	II.1	2	M	35	*GATA2*	MDS/MMS	0	Complex	NT	Complete chimerism	allo‐HSCT	20
G	II.3	2	M	32	*GATA2*	MDS/MMS	0	47,XY,+8 [5]; 46,XY [11]	NT	*Exitus*	allo‐HSCT	144
H	III.1	1	M	70	*DDX41*	MDS	1	NA	Aza	Alive	No	27
I	PN‐09	1	F	58	*DDX41*	AML	21	Complex	Clinical trial	Progression	No	9

**Table 3:** Germline variants identified in the current study. ALL, acute lymphoblastic leukemia.

Family	Individual	Cohort	Relationship	Nontumoral sample analyzed	Gene	Variant	Protein	Effect	VAF at diagnosis	Family history
A	III.1	2	Proband	CR BM	*CEBPα*	c.68dupC	p.His24AlafsTer84	Frameshift	0.51	AML, solid tumors
A	III.2		Sister	PB	*CEBPα*	c.68dupC	p.His24AlafsTer84	Frameshift		
A	II.1		Mother	CR BM	*CEBPα*	c.68dupC	p.His24AlafsTer84	Frameshift		
B	IV.2	2	Proband	CR PB	*ASXL1*	c.2110G>A	p.Gly704Arg	Missense	0.53	AML, PMF
B	III.6		Paternal Uncle	Diagnostic BM	*ASXL1*	c.2110G>A	p.Gly704Arg	Missense	0.49	
C	III.6	1	Proband	PB TL	*ETV6*	c.145C>T	p.Arg49Cys	Missense	0.49	MDS, solid tumors
C	II.6		Mother	Diagnostic BM	*ETV6*	c.145C>T	p.Arg49Cys	Missense		
C	V.2		Niece Unaffected carrier	PB	*ETV6*	c.145C>T	p.Arg49Cys	Missense		
D	II.1	1	Proband	Skin biopsy	*TP53*	c.844C>T	p.Arg282Trp	Missense	0.89	AML, ALL, solid tumors
D	II.4		Brother Unaffected carrier	PB	*TP53*	c.844C>T	p.Arg282Trp	Missense		
D	II.5		Brother Unaffected carrier	PB	*TP53*	c.844C>T	p.Arg282Trp	Missense		
E	II.2	1	Proband	PB TL	*MPL*	c.313T>C	p.Phe105Leu	Missense	0.5	MPN, thrombosis
F	II.1	2	Proband	Skin biopsy	*GATA2*	c.1187G>A	p.Arg396Gln	Missense	0.57	No
G	II.3	2	Proband	Skin biopsy	*GATA2*	c.1186C>T	p.Arg396Trp	Missense	0.49	No
H	III.1	1	Proband	PB TL	*DDX41*	c.1015C>T	p.Arg339Cys	Missense	0.52	Severe aplasia, MDS, ALL
I	PN‐09	1	Proband	Skin biopsy	*DDX41*	c.88_96delGACGAGGAC	p.Asp30_Asp32del	In frame	0.49	No

Regarding cohort 2, a total of 11 variants were detected, of which 64% (7/11) were classified as P/LP and 36% (4/11) were classified as VUS. Four variants were detected in the genes of interest with a VAF > 0.4 and were subsequently confirmed to be germline. In cohort 2, 80% (4/5) of patients were identified as carriers of disease‐causing germline variants in *CEBPα* (p.His24AlafsTer84)*, TP53* (p.Arg282Trp)*, ASXL1* (p.Gly704Arg), and *GATA2* (p.Arg396Trp) genes Tables 2 and 3.

In summary, nine patients presented MNGP Tables 2 and 3. Three patients had AML, two MDS/Mono‐MAC syndromes, two MPN, and two presented MDS. Mono‐MAC syndrome represents a unique clinical entity characterized by monocytopenia, lymphedema, an increased risk of opportunistic infections, and hematological malignancies [27].

These patients presented great heterogeneity in the age at diagnosis (median age 47 years, range 2–73).

**Fig. 2:**
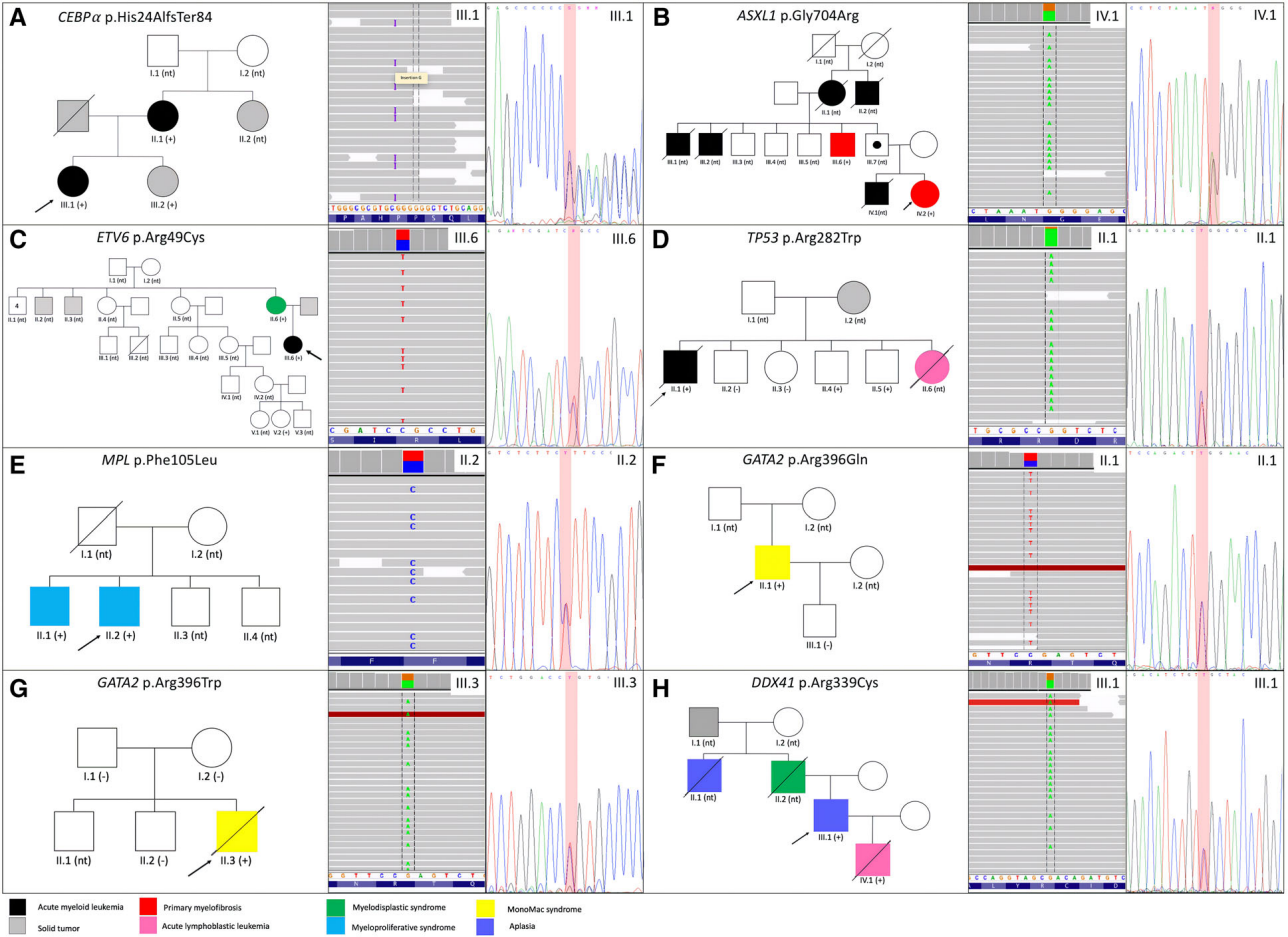
Pedigrees of patients with a confirmed MNGP. (A) AML (germline *CEBPα* p.His24Alafs). (B) MPN (germline *ASXL1* p.Gly704Arg). (C) AML (germline *ETV6* p.Arg49Cys). (D) AML (germline *TP53* p.Arg282Trp). (E) MPN (germline *MPL* p.Phe105Leu). (F) MDS/Mono‐Mac syndrome (germline *GATA2* p.Arg396Gln). (G) MDS/Mono‐Mac syndrome (germline *GATA2* p.Arg396Trp). (H) MDS (germline *DDX41* p.Arg339Cys). Arrowhead indicates index proband. Pedigree for Family I is not shown because no detailed family information was collected. nt, not tested.

The pedigrees of eight families are depicted in Fig. 2A–H. Family I is not shown since the patient denied any relevant family background, but did not give additional information about relatives to complete her pedigree.

Considering the family history of hematopoietic neoplasms or solid tumors, we would like to highlight that six out of nine patients confirmed to harbor a germline variant presented a strong family history (three patients from cohort 1 and three patients from cohort 2). Only one patient with *DDX41* germline mutation and the two cases of Mono‐Mac syndrome (*GATA2* variants) had no relevant family history (Families F, G, I; Tables 2 and 3.

Moreover, it should be remarked that family history of all 93 patients included in the study was carefully evaluated and 10 patients did present a highly suggestive family history. Among them, with the panel employed, we have been able to detect a germline alteration in 60% of the patients (6/10). The remaining four did not show any germline variant in none of the genes studied despite of strong family background (three patients from cohort 1 and one from cohort 2).

Molecular alterations detected in *CEBPα*, *TP53,* and *GATA2* have already been identified as high‐risk germline alleles with MN predisposition [27, 28, 29, 30, 31, 32],meanwhile, *ASXL1* variant p.Gly704Arg has only been found in patients diagnosed from sporadic MN and missense *MPL* variant p.Phe105Leu has been previously described in a case of chronic myelomonocytic leukemia and in a case of Sézary syndrome [33, 34]. Other missense variants (p.Pro106Leu and p.Arg102Pro) have been described as germline mutations affecting the MPL/JAK2 signaling axis in hereditary thrombocytosis [35, 36]. This new variant affects the extracellular domain of the gene, particularly a region involved in ligand binding [37]. The three *in silico* predictors classify this new variant as probably pathological. Therefore, we speculate that the nearby p.Phe105Leu mutation might have a similar disruptive capacity. Two patients harbored novel heterozygous germline variants in the *DDX41* gene (p.Asp30_Asp32del and p.Arg339Cys), one of the most frequently mutated MN predisposition gene [38]. Additionally, we have detected a germline variant in the *ETV6* gene (p.Arg49Cys) in both the index case and the mother of family J. The aforementioned alteration was found in COSMIC database as a pathogenic variant reported in a case of endometrioid carcinoma. Moreover, the prevalence of the alteration in the general population is 0.0017.

Intrafamiliar heterogeneity could suggest that the acquisition of certain somatic variants may drive the evolution of the disease. The patient A.III.1 presented a somatic variant in *GATA2* gene (p.Gly320Asp). Secondly, the subject B.III.6 acquired second‐hit *ASXL1* mutation (p.Gln976Ter). Lastly, the index case H.III.1 acquired a second‐hit *DDX41* somatic mutation (p.Ile592Phe).

## 4 Discussion

Cancer predisposition has long been recognized to contribute to the development of many solid tumors. Recently, with the incorporation of NGS, new genes have been discovered correlating with MNGP and several family studies have been carried out to date [39]. Although the field of predisposition to hematological malignancies is evolving promptly, the penetrance, the phenotype, or the inheritance pattern of this subgroup of syndromes is still unknown. Etiology of MNGP is heterogeneous, and our data reinforce this idea. In fact, there are families with a homogeneous clinical phenotype, in which all affected members present the same subtype of disease,whereas other families have a mixed phenotype, with affected members suffering from different neoplasms.

In this regard, the information obtained from family history seemed to indicate that MN in these families may be inherited in an autosomal dominant pattern with variable expression. Furthermore, the comparison of second‐generation and first‐generation patients showed a younger age of diagnosis in *CEBPα* and *ASXL1* pedigrees. These families may be possible attached to a phenomenon of anticipation.

When a genomic test is performed in patients with MN, it is crucial to confirm the germline origin of the variants found. According to our experience, cultured skin fibroblasts are the preferred source remaining as the gold standard sample for confirmation. Even though, other nontumor samples such as PB at CR, buccal swab, saliva, or isolated T cells can be used when a skin biopsy is not available or when a rapid clinical decision is needed (e.g., when there is a relative suitable as donor for allo‐HSCT) [40]. However, these samples must be interpreted carefully as they may be contaminated with tumor tissue. In remission samples, it has already been described the presence of clonal hematopoiesis of indeterminate potential which is defined as the presence of recurrent somatic mutations in hematopoietic stem cells acquired throughout the life of the patients [41]. In saliva, the presence of a significant percentage of hematopoietic cells is conceivable, and, on the other side, the variant could have been acquired in a primary stem cell and be shared between myeloid and lymphoid lineages, so it could lead to a false positive if T cells are studied for validation [42].

On the other hand, those families in which there are several relatives affected from MN and the variant is found in more than one of them, cultured skin fibroblasts are not mandatory to confirm the germline origin. Moreover, in those cases in whom the variant is detected in a remission sample or in TL there would be enough evidence to report the high suspicion of the case and provide genetic counseling to the family.

The implementation of a gene panel analysis by NGS for somatic mutations is increasingly used in the diagnosis, prognosis, and treatment selection for patients with MN [43, 44]. In this sense, the fact that known genes related to predisposition to MN are also recurrently mutated genes in *de novo* neoplasms makes plausible the identification of suspicious germline variants in the routine analysis of tumor samples [13].

In cohort 1, 32 out of 88 patients (36.4%) were suspected of carrying a germline variant since they showed pathogenic or likely pathogenic variants in any of the 15 genes with potential germline relevance. Among these suspicious patients, 21 patients were studied and discarded, six patients could not be analyzed and five patients presented germline variants. Thus, we identified an incidence of 19.3% (5/26) in suspicious patients and a final incidence of confirmed MNGP of 6% (5/82). Our results are consistent with previous studies where it is estimated that around a 5% of all cancers present a hereditary component [13, 45, 46].

This percentage is increased when patients with highly suggestive family background (*n* = 10) are taken into account. In this situation, our panel was able to detect the causative mutation in 60% of the patients (*n* = 6).

Furthermore, the panel employed was capable to reveal a germline variant in three cases from cohort 1 where family history was not known or suspected. Due to the fact that some relevant genes are missing from the panel, the number of patients identified may even be underestimated. That is why our gene panel will be updated with a new enhanced version which will include, *inter alia*, *SAMD9, SAMD9L,* or *SRP72* genes. The inclusion of genes associated with hereditary myeloid malignancies into routine myeloid panels will reduce costs and optimize patient care. However, in this scenario, it is imperative to standardize which variants should be reported and pretest counseling. Additionally, genetic analysis may become even more challenging since some polymorphisms are yet known to be associated with an increased risk of developing cancer [47, 48]. Nevertheless, it still needs to be established the role of common genetic variants in cancer predisposition syndromes.

Those individuals with a highly suggestive family history but no variant found are interesting candidates to enlarge the genetic study with other available approaches such as a panel of cancer predisposition genes, whole‐exome sequencing (WES) or whole‐genome sequencing (WGS), in order to identify the causal alteration. In the case of WES/WGS, at least two affected members of the family must be analyzed to discover which are the genes involved.

Thus, family history is so relevant that we consider it crucial to incorporate specific personal medical data of all patients diagnosed with MN in order to collect relevant family history for clinical suspicion. However, variable expression and incomplete penetrance of these disorders add imperative challenges [49]. That is why it is important to carefully analyze the suspicious variants detected through NGS in order to provide the best clinical care, guide stem cell transplant decision, and identify other relatives in the family who may be at risk.

The identification of variants which confer a predisposition to the development of hematological malignancies is challenging because as more pedigrees are described, surely one variant will be associated with a unique family [1, 50].

We herein report a missense *ASXL1* mutation (p.Gly704Arg) which, to our knowledge, has not been previously described as a germline variant. It has only been previously found in patients diagnosed with sporadic MN [29, 51]. To date, few patients with MNGP have been reported to present germline *ASXL1* mutations [16, 52], however, the strong AML family history found within this family together with the fact that the variant was detected in a remission sample from the proband (B.IV.3), as well as in a diagnosis sample from her paternal uncle (B.III.6), supports the idea of its pathogenicity and germline origin Fig. 2. It must be highlighted that these data do not replace the need for further experimental validation, albeit it is reasonable to consider this variant as a predisposing risk allele in our family. We also report on a new missense germline variant in the *MPL* gene (p.Phe105Leu). It affects the extracellular domain of the gene, particularly a region involved in ligand binding [37] and a disruptive capacity was predicted. Further functional studies are required to determine the pathogenic potential of those non described variants and the significance of them in inherited related MN.

Both germline variants in the *DDX41* gene are located throughout the sequence of the gene, suggesting that different mutations can impact on different protein domains or motifs with pathologic consequences [38]. Despite small sample size, one out of 2 (50%) patients with germline *DDX41* mutations in the present study harbored somatic point mutations in the other allele, as previously reported [53]. Finally, the precise function of the novel *ETV6* mutation is unknown, although it is located in exon 2 and, in the protein level, near the SAM‐PNT domain which mediates protein–protein interaction with Ets factors [54]. This p.Arg49Cys *ETV6* variant has been previously described by Moriyama *et al*. [55] in a cohort of childhood acute lymphoblastic leukemia, revealing the complexity of hereditary cancer as specific mutations can confer different risks to different types of cancer [56].

When allo‐HSCT is considered in a patient harboring a germline variant, donor selection among relatives must be carefully conducted. Potential‐related donors should be evaluated for the mutation detected in the index case in order to avoid selecting an asymptomatic carrier as donor (42). In those cases in which the donor presents the alteration, alternative donors should be considered. In our cohort, patients D.II.1, F.II.1, and G.II.3 underwent allo‐HSCT. For all of them, a screening of the mutation was performed in the candidate family members and donors were selected among those who did not carry the alteration. In the case of family D, despite the fact that the patient died before receiving the transplant, the alteration was detected in several siblings Table 3, Fig. 2. All of them are still at risk for suffering from hematological and solid tumors, since the alteration detected in *TP53* gene (p.Arg282Trp) is diagnostic for the Li–Fraumeni syndrome [28, 32].

Within this scenario, genetic counseling should be offered to family members. Healthy family members diagnosed with a hematological predisposition syndrome should be referred to cancer surveillance programs. The optimal clinical surveillance for asymptomatic individuals with germline alterations is unclear. Germline alterations are no fully penetrant, and many carriers will not develop any malignancy. In accordance with other authors, analysis of complete PB counts every 6 months is highly recommended. If there is any change in the blood counts, the test must be repeated 1–2 weeks later, and if it persists, it will be necessary to perform a BM aspirate [57]. However, if the syndrome is associated with the onset of other tumors, this screening will be insufficient. Therefore, it is necessary to implement a surveillance specific program for each of the hematological hereditary syndromes and their follow‐up should be performed by a multidisciplinary team in specialized centers.

Research in this area is necessary to characterize in detail each of these syndromes, as well as the establishment of national and international registries. In the same way, it is crucial for clinicians to be familiar with these syndromes and they should keep in mind hematological malignancy predisposition syndromes on the differential diagnosis for every patient in order to identify properly families with predisposing conditions [10], [58].

## 5 Conclusions

In conclusion, we demonstrated that it is useful to include genes related to MNGP in gene panels designed principally for routine analysis of somatic mutations. They provide an effective approach to: (a) detect clinical significant variants of potential germline origin, and therefore (b) avoid using a related stem cell donor carrying the same mutation, and (c) offer genetic counseling to the families affected.

In the future, the number of patients diagnosed with germline alteration will certainly increase as the full spectrum of genes involved in hematological malignancies syndromes is elucidated. This issue would be solved since WES, or even WGS, techniques will become a more cost‐effective and timely approach in routine clinical care.

## Conflict of interest

The authors declare no conflict of interest.

## Author contributions

CA‐Z, JS‐G, IB, CM‐L, and JLD‐M conceptualized the study; CA‐Z, JS‐G, DC, MC, and PM curated the data; GR‐M, ND, SO, PF, MB, and MK recorded patient clinical characteristics; CA‐Z, JS‐G, DC, MC, PM, IB, and CM‐L involved in formal analysis; JLD‐M, IB, and CM‐L involved in funding acquisition; JS‐G, IB, and CM‐L supervised the study; CA‐Z, JS‐G, IB, and CM‐L wrote—original draft; CA‐Z, JS‐G, GR‐M, ND, SO, PF, DC, MC, PM, MB, MK, JLD‐M, IB, and CM‐L wrote—review and editing.

## Supporting information

**Fig. S1.** Evidence framework and criteria for classifying pathogenic variants according to the recommendations of the American College of Medical Genetics and Genomics and the Association for Molecular Pathology.
**Table S1.** Genes and recurrent alterations included in the NGS gene panel employed.
**Table S2.** Confirmatory analysis for suspicious variants in the genes of interest with a VAF > 0.4 in cohort 1.
