# Supplementary material for: Clinical utility of targeted next‐generation sequencing for the diagnosis of myeloid neoplasms with germline predisposition
Source: Mol Oncol. 2021 Jul 16;15(9):2273–84. doi: 10.1002/1878-0261.12921 (PMC8410541; doi:10.1002/1878-0261.12921)
Supplement: Supplementary file 1 — Fig. S1. Evidence framework and criteria for classifying pathogenic variants according to the recommendations of the American College of Medical Genetics and Genomics and the Association for Molecular Pathology. Table S1. Genes and recurrent alterations included in the NGS gene panel employed. Table S2. Confirmatory analysis for suspicious variants in the genes of interest with a VAF > 0.4 in cohort 1. [file MOL2-15-2273-s001.pdf]

Clinical utility of targeted next generation sequencing for the diagnosis of myeloid neoplasms with germline predisposition

Cristina Andrés-Zayas[1,2*], Julia Suárez-González[1,2*], Gabriela Rodríguez-Macías[3], Nieves Dorado[2,3], Santiago Osorio[2,3], Patricia Font[2,3], Diego Carbonell[2,3], María Chicano[2,3], Paula Muñiz[2,3], Mariana Bastos[2,3], Mi Kwon[2,3], José Luis Díez-Martín[2,3,4], Ismael Buño[1,2,3,5**], Carolina Martínez-Laperche[2,3,#,**].

[1]Genomics Unit, Gregorio Marañón General University Hospital, Gregorio Marañón Health Research Institute (IiSGM), Madrid, Spain.

[2]Gregorio Marañón Health Research Institute (IiSGM), Madrid, Spain.

[3]Department of Hematology, Gregorio Marañón General University Hospital, Madrid, Spain.

[4]Department of Medicine, School of Medicine, Complutense University of Madrid, Spain.

[5]Department of Cell Biology, School of Medicine, Complutense University of Madrid, Spain.

(*) CA-Z and JS-G contributed equally to this work. (**) IB and CM-L contributed equally to this work.

Running Title: Germline mutations in myeloid neoplasms by NGS

Conflict of interest statement: The authors have nothing to disclosure

[#]Corresponding Author:

Carolina Martínez-Laperche, PhD

Hematological Genetics Laboratory, Department of Hematology.

Gregorio Marañón General University Hospital

Gregorio Marañón Health Research Institute (IiSGM)

C/ Doctor Esquerdo 46

28007 Madrid

TEL: 915868775

FAX: +34915868394

E-mail: cmlaperchehgugm@gmail.com

Suplementary Table 1

Single nucelotide variants, small insertion and small deletions		
**Gene**	**Gene-ID**	**Exon**
NPM1	ENSG00000181163	12
FLT3	ENSG00000122025	20,14,15
DNMT3A	ENSG00000119772	7,23
IDH1	ENSG00000138413	4
IDH2	ENSG00000182054	4
CEBPa	ENSG00000245848	1
KIT	ENSG00000157404	8,17
TET2	ENSG00000168769	All
RUNX1	ENSG00000159216	3-8
ASXL1	ENSG00000171456	12,13
TP53	ENSG00000141510	2-11
WT1	ENSG00000184937	7,8,9
NRAS	ENSG00000213281	2-3
KRAS	ENSG00000133703	2,3,4,5
SRSF2	ENSG00000161547	1
JAK2	ENSG00000096968	8,12,13,14,15
MPL	ENSG00000117400	3,4,5,6,10,12
CALR	ENSG00000179218	9
CSF3R	ENSG00000119535	6,7,8,14,15,16,17
SETBP1	ENSG00000152217	4
ZRSR2	ENSG00000169249	All
SF3B1	ENSG00000115524	12,13,14,15,16
EZH2	ENSG00000106462	All
PTPN11	ENSG00000179295	3,4,13
ABL1	ENSG00000097007	4-11
ETNK1	ENSG00000139163	3
CBL	ENSG00000110395	4,5,8,9,10,11
NF1	ENSG00000196712	All
DDX41	ENSG00000183258	All
ANKRD26	ENSG00000107890	All, 5'UTR
ETV6	ENSG00000139083	All
GATA2	ENSG00000179348	3,4,5,6,7
VHL	ENSG00000134086	2,3
EPOR	ENSG00000187266	All
IKZF1	ENSG00000185811	2,3,4,5,6,7,8

Traslocations			
Traslocation	**Fusion gene**	**Gene ID-1**	**Gene ID-2**
t (9;22)(q34;q11.2)	*BCR-ABL1*	ENSG00000186716	ENSG00000097007
t (5;var)(q31-q33,var)	var-PDGFRb	ENSG00000113721	-
t (8;var) (p11,var)	var-FGFR1	ENSG00000077782	-
t(8;21)(q22;q22)	*RUNX1-RUNX1T1*	ENSG00000159216	ENSG00000079102
t(16;16)(p13.1;q22)//inv(16)(p13;q22)	*CBFB-MYH11*	ENSG00000067955	ENSG00000133392
t(15;17)(q22;q21)	*PML-RARa*	ENSG00000140464	ENSG00000131759
t(17;var) (q21)	*RARA-var*	ENSG00000131759	-
t(11;var)(q23;var)	var-KMT2A	ENSG00000118058	-
t(6;9)(p23;q34)	*DEK-NUP214*	ENSG00000124795	-
t(1;22)(p13;q13)	*RBM15-MKL1*	ENSG00000162775	-
t(8;9)(p22;p24)	*PCM1-JAK2*	ENSG00000078674	ENSG00000096968

Structural alterations				
**Large Deletions**	**Region**	**Gene**	**Gene ID-1**	**Gene ID-2**
del4q12 (CHIC2)	*FIP1L1-PDGFRA*	*FIP1L1-PDGFRA*	ENSG00000145216	ENSG00000134853
del 17p13.1 (TP53)	17p13.1 (TP53)	TP53	ENSG00000141510	
del(7q)	7p11.2	IKZF1	ENSG00000185811	
	7q21.12	*CDK6*	ENSG00000105810	
	7q22.1	*CYP3A5*	ENSG00000106258	
	7q31.2	*CAV1*	ENSG00000105974	
	7q36	*EZH2*	ENSG00000106462	
del(5q)	5p13.2	*NIPBL*	ENSG00000164190	
	5q22.2	*APC*	ENSG00000134982	
	5q31.2	*EGR1*	ENSG00000120738	
	5q32-5q33	*TCOF1*	ENSG00000070814	
	5q33.1	*ATOX1*	ENSG00000177556	
del(11q)	11q23.3	*KMT2A*	ENSG00000118058	
	11q24.2	*HEPN1*	ENSG00000221932	
	11q24.3	*ST14*	ENSG00000149418	
del(12p)	12p13.1	*GRIN2B*	ENSG00000273079	
	12p13.2	*ETV6*	ENSG00000139083	
del(20q)	20q11.21	*ASXL1*	ENSG00000171456	
	20q11.23	*SRC*	ENSG00000197122	
	20q13.12	*SULF2*	ENSG00000196562	
	20q13.12	*STK4*	ENSG00000101109	
**Aneusomy**	**Region**	**Gene**	**Gene ID-1**	
+8	8p12	*NRG1*	ENSG00000157168	
	8q13.3	*PRDM14*	ENSG00000147596	
	8q21.3	*NBN*	ENSG00000104320	
	8q24.21	*MYC*	ENSG00000136997	
	8q24.3	*CYP11B2*	ENSG00000179142	
+19	19q13.11	*CEBPα*	ENSG00000245848	

Supplementary Table 1. Genes and recurrent alterations included in the NGS gene panel employed.

Suplementary Table 2

Gene	Transcript	Variant	Protein	VAF	Germline analysis	Sample
GATA2	NM_032638.5	c.1423A>T	p.Met475Leu	0.57	Negative	RBM
TP53	NM_001126112.2	c.470dupT	p.Arg158ProfsTer23	0.41	Negative	RBM
RUNX1	NM_001754.5	c.958C>T	p.Arg320Ter	0.42	Negative	RPB
TP53	NM_001126112.2	c.838A>G	p.Arg280Gly	0.49	Negative	RBM
GATA2	NM_032638.5	c.989G>A	p.Arg330Gln	0.41	Negative	FB
CEBPa	NM_004364.4	c.971T>G	p.Leu324Arg	0.47	Negative	FB
RUNX1	NM_001754.5	c.305dupT	p.Pro103AlafsTer35	0.52	Negative	RPB
CBL	NM_005188.4	c.1259G>A	p.Arg420Gln	0.92	Negative	FB
CSF3R	NM_000760.4	c.2503G>A	p.Glu835Lys	0.47	Negative	RPB
TP53	NM_001126112.2	c.451C>T	p.Pro151Ser	0.81	Negative	RPB
CEBPa	NM_004364.4	c.59_60insTC	p.Gln20HisfsTer141	0.48	Negative	RBM
NF1	NM_001042492.3	c.910C>T	p.Arg304Ter	0.45	Negative	RPB
TP53	NM_001126112.2	c.637C>T	p.Arg213Ter	0.70	Negative	TL
CEBPa	NM_004364.4	c.209delC	p.Pro70ArgfsTer90	0.44	Negative	RBM
GATA2	NM_032638.5	c.1052A>T	p.Asn351Ile	0.49	Negative	RBM
RUNX1	NM_001754.5	c.1267_1271delCGCTC	p.Arg423AlafsTer175	0.54	Negative	RBM
CEBPa	NM_004364.4	c.912_920delGCAGCGCAA	p.Lys304_Arg306del	0.43	Negative	RBM
RUNX1	NM_001754.5	c.305dupT	p.Pro103AlafsTer35	0.52	Negative	RPB
CSF3R	NM_000760.4	c.2503G>A	p.Glu835Lys	0.55	Negative	RPB
MPL	NM_005373.3	c.313T>C	p.Phe105Leu	0.52	Positive	RPB
GATA2	NM_032638.5	c.1187G>A	p.Arg396Gln	0.57	Positive	FB
DDX41	NM_016222.4	c.1015C>T	p.Arg339Cys	0.45	Positive	TL
DDX41	NM_016222.4	c.88_96delGACGAGGAC	p.Asp30_Asp32del	0.52	Positive	TL
ETV6	NM_001987.5	c.145C>T	p.Arg49Cys	0.49	Positive	TL
ASXL1	NM_015338.6	c.3306G>T	p.Glu1102Asp	0.49	Negative	RBM
ASXL1	NM_015338.6	c.2564_2567delATTG	p.Asp855AlafsTer11	0.48	Negative	RBM
JAK2	NM_004972.4	c.1849G>T	p.Val617Phe	0.56	Negative	RPB

Supplementary Table 2. Confirmatory analysis for suspicious variants in the genes of interest with a VAF>0.4 in cohort 1. Abbreviations: RPB: remission peripheral blood; RBM: remission bone marrow; TL: T lymphocytes; FB: fibroblasts

Suplementary Figure 1

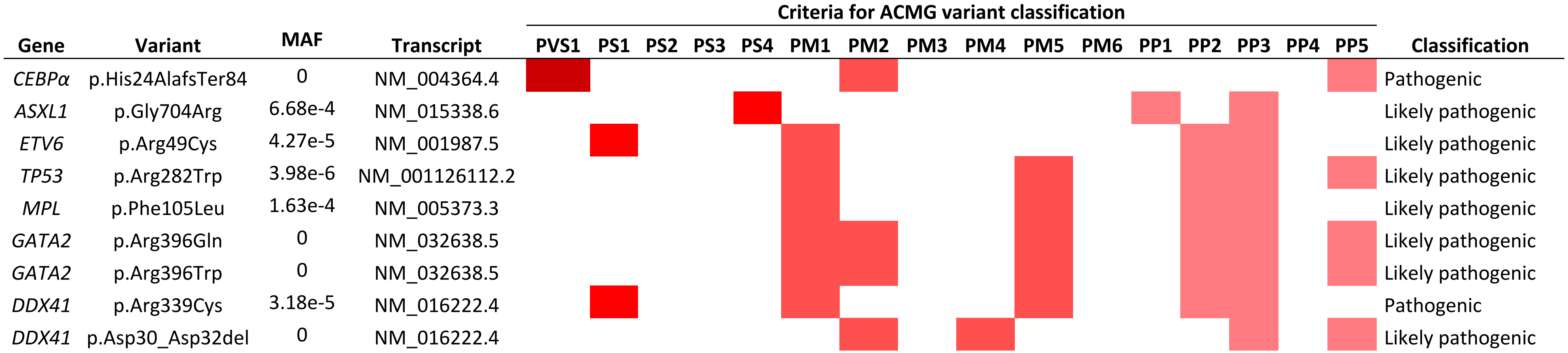

Supplementary Figure 1. Evidence framework and criteria for classifying pathogenic variants according to the recommendations of the American College of Medical Genetics and Genomics and the Association for Molecular Pathology. Abbreviations: MAF: Minor Allele Frequency; PVS: pathogenic very strong; PS: pathogenic strong; PM: pathogenic moderate; PP: pathogenic supporting.
